# Utilization of the directional balloon technique to improve the effectiveness of percutaneous kyphoplasty in the treatment of osteoporotic vertebral compression fractures and reduction of bone cement leakage

**DOI:** 10.1097/MD.0000000000015272

**Published:** 2019-05-13

**Authors:** Pu Wang, Jin Li, Zukun Song, Zhan Peng, Guangye Wang

**Affiliations:** Department of Spinal Surgery, Affiliated Baoan Hospital of Shenzhen, Southern Medical University, Shenzhen, China.

**Keywords:** bone cement leakage, directional balloon, osteoporotic vertebral compression fracture (OVCF), percutaneous kyphoplasty (PKP)

## Abstract

**Background:**

This article evaluates the effectiveness of a new directional balloon technique in the treatment of osteoporotic vertebral compression fractures (OVCFs).

**Methods:**

From September 2015 to April 2017, 100 patients with single-segment OVCFs treated using percutaneous kyphoplasty were available for complete data assessment. Among these, 51 cases were treated with the traditional nondirectional balloon technique (group 1) and 49 cases were treated with the directional balloon technique (group 2). Operative time, blood loss, and X-ray exposure time were compared between the 2 groups. The visual analogue score (VAS), Oswestry disability index (ODI), and Roland Morris disability (RMD) scores, and wedge-shaped and kyphosis angles were measured at 3 days and 1, 3, 6, and 12 months, respectively, after surgery.

**Results:**

There were no significant differences in blood loss or the amount of bone cement injected between the 2 groups; however, operative times, X-ray exposure times, and leakage rates of bone cement, especially type C in group 2, were significantly lower in group 2 than those in group 1. VAS, ODI, and RMD scores, and wedge-shaped and kyphosis angles at each time point after surgery were significantly higher than those before surgery. However, the improvement in VAS, ODI, and RMD scores in group 2 was only significantly better than those in group 1 at 3 days after surgery.

**Conclusion:**

The utilization of the directional balloon technique in the treatment of OVCFs using percutaneous kyphoplasty can not only reduce the operation time, the radiation, and the bone cement leakage, but also improve the early curative effect.

## 1 Introduction

The main symptoms of osteoporotic vertebral compression fractures (OVCFs), which have high incidence in the elderly, are localized pain in the waist, limited daily activity, reduced self-care ability, and depression. Mobility disability and reduced balance caused by OVCFs have resulted in high morbidity,^[1]^ which has gradually become a major health problem worldwide.^[2]^

The short-term goals of treating OVCFs are to relieve pain, restore stability, and improve activity. To achieve these goals, patients may receive conservative treatment such as anesthetic analgesics, bed rest and so on, or undertake a vertebral augmentation procedure (VAP).^[3]^ However, drug therapy is not always effective and can be painful, and prolonged bed rest aggravates osteoporosis and can lead to fatal complications such as pneumonia, atelectasis, and deep vein thrombosis.^[4]^ Therefore, VAP has been widely accepted as the preferred treatment by many patients suffering from OVCFs.

Vertebroplasty was designed to prevent the fretting of the fracture area and to restore the strength of the fracture body by injecting bone cement into the vertebral body, thereby reducing pain and preventing further collapse of the vertebral body.^[5]^ Kyphoplasty involves the area to be injected receiving predilation by a balloon before injection of the bone cement, which restores vertebral height and prevents bone cement leakage by low pressure injection. VAP is considered to be a relatively safe and minimally invasive technique that can relieve pain quickly; however, some complications are still inevitable.^[6]^ Bone cement leakage is one of its most common complications because it damages the spinal cord when leakage into the spinal canal occurs and can lead to pulmonary embolism when leakage to the pulmonary vein occurs. The consequences of both these situations can be disastrous.^[7]^ Therefore, several researchers had attempted to improve surgical techniques and instruments to allow the bone cement to be injected more effectively into the vertebral body, thus minimizing any bone cement leakage.^[8–11]^

The balloon dilator that is widely utilized during percutaneous kyphoplasty (PKP) is designed with nondirectional expansion, which dilates along the direction of minimum resistance. If the puncture position is not ideal, such as near the cortical bone or endplate of the fractured vertebral body, then the expanded balloon might burst the vertebral body, causing iatrogenic fractures and further bone cement leaks. By controlling the direction of the balloon expansion, the surgeon can inject the bone cement more effectively, reducing bone cement leakage. The objective of the present study was to evaluate the value of a new directional balloon technique in the treatment of OVCFs with PKP via a random prospective study.

## 2 Materials and methods

### 2.1 Patients and study design

We conducted a prospective, randomized case-controlled study in Affiliated Baoan Hospital of Shenzhen, Southern Medical University, between September 2015 and April 2017.

All patients aged 60 years or above with severe back pain and limited movement due to acute OVCFs, and who could not tolerate conservative therapy in the emergency room or outpatient clinic were included in the study. Every included patient was given a serial number according to the consecutive sequence of recruitment, and randomly assigned to conventional balloon dilation technology (group 1) or directional balloon dilation technology group (group 2) using computer-generated randomized codes, according to the serial number. All allocation information was concealed in numbered opaque, sealed envelopes. Patients and practitioners were all blinded to the randomization assignment and allocation.

Inclusion criteria included patients with single-segment fresh OVCF with complete rear wall occurring within 2 weeks of acute minor or mild trauma (confirmed by computed tomography scan and T2-weighted short-tau inversion recovery sequences by magnetic resonance imaging); 5 scores or more in the visual analogue score (VAS) of back pain [determined to be osteoporosis or severe osteoporosis with decreased bone mineral density (T < −2.5 standard deviation)]; no serious cardiopulmonary disease; and patients who could tolerate surgery.

Exclusion criteria included patients diagnosed with peritraumatic chronic back pain, degenerative scoliosis, and injured posterior wall insufficiency; and severe cardiopulmonary dysfunction, disorder of coagulation mechanism, systemic infection, local osteomyelitis, vertebral compression fractures caused by malignant tumor, nerve root pain, or spinal cord compression syndrome.

**Figure 1:**
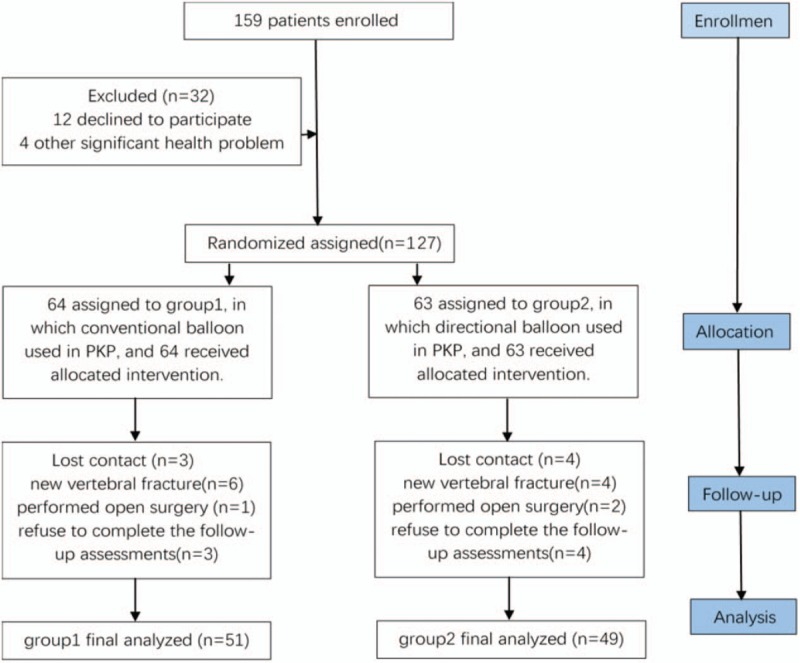
The trial profile.

The study was approved by the Ethics Committee of Affiliated Baoan Hospital of Shenzhen, Southern Medical University, Shenzhen, China, and patients provided written informed consent before participation. The trial profile is shown in Figure 1, with demographic information about the patients who were present at final follow-up (at 12 months) given in Table 1.

**Table 1:**
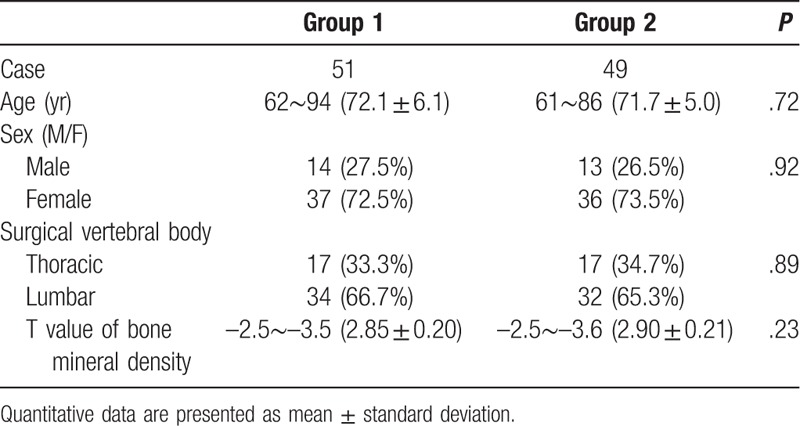
Comparison of the demographics between 2 groups in this study.

### 2.2 Preoperative treatment

Strict bed rest and bracing treatment were required for all patients. Postural reduction was also performed before surgery.

### 2.3 Operative technique

Local anesthesia was administered during the operation. After the patients were located in the prone position, the puncture needle was inserted from the more severe side under the guidance of a C-arm machine guided by unilateral puncture. When the needle tip reached 3 to 4 mm in the posterior edge of the vertebral body, the needle core was pulled out and the working sleeve retained in the vertebral body. A drill was then inserted into the working channel and drilled into the vertebral body. When the fine drill reached 3/4 of the vertebral body and reached or crossed the midline of the vertebral body, it was removed. A balloon was then inserted into the vertebral body through the working cannula. Then, 4 mL of contrast agent was inserted into the balloon under C-arm X-ray fluoroscopy to dilate the balloon and reduce the fracture. When the fracture was satisfactorily reduced, the balloon was withdrawn and low viscosity bone cement was injected into the vertebral cavity. When the surgeon judged that the vertebral body was satisfactorily filled or that the cement had been diffused close to the posterior wall of the vertebral body, injection of cement stopped, a sufficient time was allowed to pass, and confirmation given that the cement was cured. Then, the cannula was rotated and pulled out.

**Figure 2:**
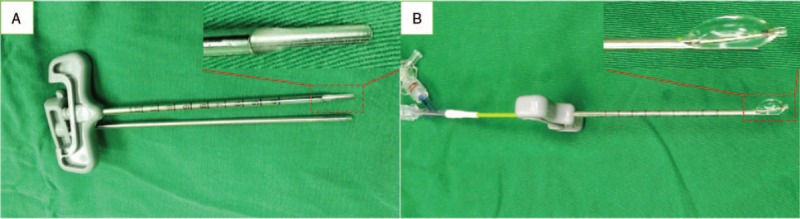
(A) The end of the working channel of directional percutaneous kyphoplasty system is designed to be notched. (B) It works in vitro. When balloon dilation was carried out, it expanded in the direction toward the notch.

**Figure 3:**
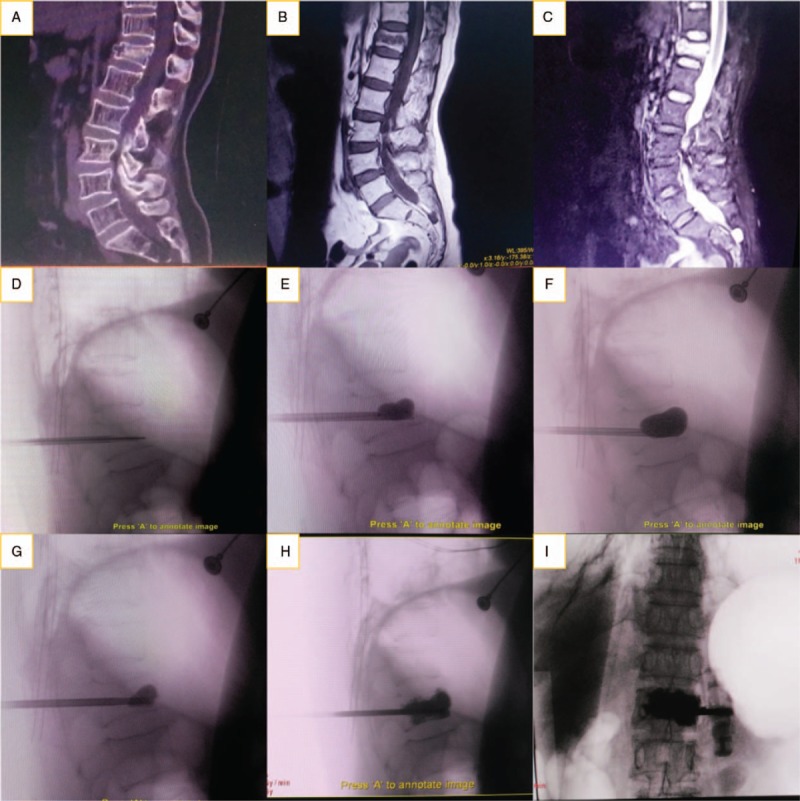
(A–C) MRI images showed a patient diagnosed with a T12 vertebral compression fracture. PKP was performed with directional balloon dilation technology. Preoperative CT, T1, and T2 fat suppression sequences of preoperative MRI showing that T12 was fresh OVCF and the fracture area was in the middle. (D–F) Images showing the needle located in the lower part of the vertebra, near the lower endplate, working channel adjusted to move the notch facing upward. In the process of balloon dilation, it expanded in an upward direction. (G–I) Images showing the cement first diffusing evenly upward and the fracture and surrounding area fully filled finally. CT = computed tomography, MRI = magnetic resonance imaging, OVCF = osteoporotic vertebral compression fracture, PKP = percutaneous kyphoplasty.

Two methods were used to dilate the balloon, reduce the fracture, and inject the bone cement. In group 1, a conventional balloon dilation technology was used (Percutaneous Kyphoplasty System, Shandong Dragon Crown Medical Co., Ltd) and in group 2, a new type of directional balloon dilation technology (Directional Percutaneous Kyphoplasty System, Suzhou and Science & Technology Development Co., Ltd) was used. The latter system has a working channel that is designed based on the traditional PKP working channel. The end of the directional balloon-working channel is notched and its edge is circular and obtuse. The working channel notches are composed of arc and slope structures. The end of the channel is approximately 1/3 arc structure in the 1 cm range, and the slope and arc structure are in the 1 to 2 cm range, and gradually transition to a circular tubular channel. To control the direction of balloon expansion, the channel gap was oriented to dilate by rotating the working channel. When the cement was injected into the vertebral body, the cement diffused in the notched direction (as is shown in Figs. 2 and 3).

### 2.4 Postoperative treatment

The patients were supine and observed for 6 h after surgery. The lumbar dorsalis muscle was exercised on the 1st day after surgery. Two days after surgery, the patients got out of bed with the protection of a brace and were protected for 6 to 8 weeks. Routine treatments of antiosteoporosis were performed after surgery and any underlying diseases were simultaneously treated.

### 2.5 Bone cement leakage

Bone cement leakage was determined by 2 or more uninformed specialists from the examination of X-rays separately. Based on the method described by Yeom et al,^[12]^ bone cement leakage was classified into 3 types: type B – cement leakage along the vertebrobasilar vein to the posterior border of the vertebral body, type C – cement leakage mainly along the cortical defect to the intervertebral disc, and type S – cement leakage mainly around the vertebral body along the intervertebral vein.

Secondary parameters operation time, blood loss, injected cement leakage, X-ray exposure time, and bone cement leakage during surgery were recorded. The VAS^[13]^ was used for pain scoring, the Oswestry Disability Index (ODI)^[14]^ was applied for functional assessment, and the physical function items in the Roland Morris disability (RMD)^[15]^ questionnaire were used for quality-of-life evaluation before surgery and at 3 days, 1 month, 3 months, 6 months, and 12 months after surgery.

Anterior–posterior and lateral spinal radiographs were obtained at 3 days, 1 month, 3 months, 6 months, and 12 months after surgery. If a new fracture was doubted, a magnetic resonance imaging was undertaken to confirm it, and it was then excluded. The wedge-shaped angle of the injured vertebra and the kyphosis angle of the upper and lower adjacent vertebrae (including 3 segments of the vertebral body) were measured using the method proposed by Pradhan et al.^[16]^

### 2.6 Statistical methods

Quantitative results are presented as means ± standard deviation. The t-test (paired t-test was used for intragroup comparison and independent sample t-test was used for intergroup comparison) and χ^2^ test (or Fisher exact test) were performed to analyze the variables using SPSS 17.0 software (SPSS Inc, Chicago, IL). According to the previous retrospective study, it was assumed that the proportion of bone cement leakage was 5% in the directional balloon group and 23% in the control group. When the sample size of the directed balloon group and the control group were both 54 cases, which was calculated using PASS software (version 11.0; NCSS, LLC), the significant difference could be detected with more than 80% power. When the loss ratio was assumed to be 10%, the number of cases per group was 60. Finally, 127 patients were included in this study. For all tests, the level of significance was set at *P* < 0.05.

## 3 Results

As shown in Figure 1, from September 2015 to April 2017, a total of 159 patients were enrolled in the present study, with 127 patients randomly divided into 2 groups. A total of 64 patients were treated with the conventional balloon technique (group 1) and 63 patients with the directional balloon technique (group 2). At the 1-year follow-up after surgery, 100 out of 127 patients were available for clinical review. The failure in follow-up of 27 patients was due to loss of contact (3 in group 1 and 4 in group 2), new vertebral fracture (6 in group 1 and 4 in group 2), performing open surgery (1 in group 1 and 2 in group 2), and refusal to complete the follow-up assessments (3 in group 1 and 4 in group 2). Thus, 51 patients in group 1 and 49 patients in group 2 were available for complete data assessment. There were no significant differences in age, sex ratio, surgical vertebral body, and bone mineral density between the 2 groups (Table 1).

### 3.1 Primary outcome

The results in Table 2 show that total bone cement leakage rate was significantly lower in group 2 than that in group 1, and the rate of the type C for bone cement leakage in group 2 was also significantly lower in group 2 than that in group 1.

**Table 2:**
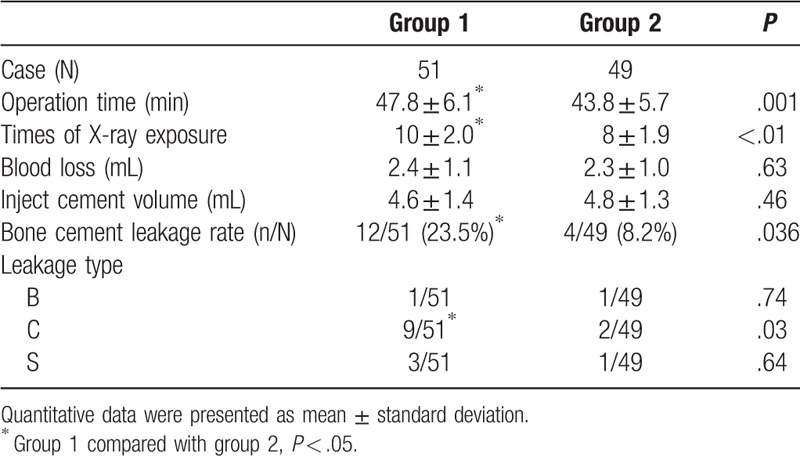
Comparison of intraoperative data between 2 groups.

### 3.2 Secondary outcomes

There were no significant differences in the injected cement volume and blood loss between the 2 groups; however, the operative times and the X-ray exposure times were significantly lower in group 2 than those in group 1. As shown in Tables 3 and 4, VAS, ODI, and RMD scores, and wedge-shaped and kyphosis angles in the 2 groups were significantly higher after surgery at 3 days, 1 month, 3 months, 6 months, and 12 months than those before surgery. The improvement in VAS, ODI, and RMD scores in group 2 were only significantly better than those in group 1 at 3 days after surgery. Similarly, the wedge-shaped angles and kyphosis angles in group 2 were also only significantly better than those in group 1 at 3 days after surgery.

**Table 3:**
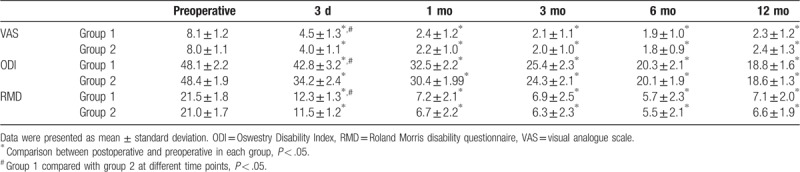
Comparison of the clinical outcomes results between 2 groups before and after operation.

**Table 4:**
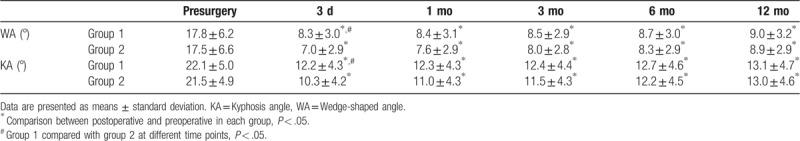
Comparison of wedge-shaped angle and kyphosis angle between the 2 groups before and after surgery.

## 4 Discussion

OVCF is a major public health problem. In the United States and Europe, it is estimated that 1.7 million such fractures occur annually.^[17]^ Since 1990, when percutaneous vertebroplasty (PVP) was first utilized by Deramond for the treatment of OVCFs,^[18]^ this technique has been quickly and widely accepted by patients because it causes less trauma, has fewer complications, and ensures a satisfactory curative effect.^[19]^ PKP was subsequently improved from PVP and is now widely utilized. Compared with PVP, PKP dilates the compressed vertebral body with a bone dilation system and forms a cavity in the vertebral body to enable the bone cement to be infused at lower pressure, which diffuses around the cavity and ensures that its distribution in the vertebral body is more uniform.^[20]^ PKP can correct the secondary spinal angulation deformity and increase the compression height of the affected vertebrae. Simultaneously, because of the low pressure of the bone cement perfusion, the rate of extravertebral leakage is significantly reduced.^[21]^ However, there is still a high rate of cement leakage in PKP.^[22]^ Thus, several improved surgical techniques and instruments have been developed to allow bone cement to be fully dispersed in the fracture area, while minimizing the leakage rate of bone cement.^[8,9,11]^ Boszczyk et al^[11]^ applied the lateral pedicle approach to vertebroplasty and discovered several new complications such as difficulty in choosing the puncture point and vascular injury. Figueiredo et al^[9]^ controlled the injection direction of bone cement and achieved directional pouring of the bone cement using a bone cement push rod with a lateral opening, in which the leakage rate of the bone cement was 27.3%. This was far lower than that of 68% when using a traditional booster, but still higher than that of the PKP group (16.7%), which might be related to its lack of low-pressure perfusion.

Considering the important role of the vertebral balloon distractor in correcting secondary spinal angulation deformities and reducing the pressure of bone cement perfusion,^[18]^ in the present study, we attempted to use a controllable balloon distractor to treat OVCFs and to achieve a lower rate of bone cement leakage while ensuring curative effect. The so-called directional balloon technique works via dilating the balloon, after which a continuous directional initial resistance is provided that causes the bilateral resistance of the balloon to become unbalanced. The initial side resistance of the balloon dilation is to bone and the resistance of the other side is the sum of the bone and metal supporting points. Thus, the balloon expands along its side with little resistance, achieving the purpose of directional expansion. To exclude the influence of other factors, we selected single-segment fresh OVCF patients with surgical indications for the present study.

In the present study, there was 1 case of type B leakage, 9 cases of type C leakage, and 3 cases of type S leakage in group 1. In comparison, the rate of bone cement leakage, especially type C, in group 2 was significantly lower than that in group 1. The reason for bone cement leakage is that the cement requires a certain degree of pressure when it is injected into the vertebral body and the cement must be fluid during the drawing period. This leads to the leakage of bone cement around the vertebral body, possibly via vertebral fracture, defective or vertebral venous plexus.^[23]^ When the balloon is too close to the upper and lower endplate, the cement easily causes leakage via the cortical approach. Based on the analysis of preoperative coronal and sagittal images utilized to judge the integrity of the endplate, the insertion point and balloon expansion direction were designed to control the reduction direction of the fracture and to protect the bone mass under the endplate, thus reducing the leakage of bone cement, especially type C leakage. Although type C cement leakage does not generally cause clinical symptoms,^[24]^ the infiltration of bone cement into the disc will affect the absorption and metabolism of the nutritional components of the disc, resulting in weakening of the disc absorption load and scattered stress function, which reduces the stability of the spine and increases the risk of fracture of the adjacent vertebral body after surgery.^[25]^ Fei et al^[26]^ obtained the same conclusion using the spinal finite element model.

Results showed that the directional balloon shortened the operation time and reduced X-ray exposure, which can be explained by the following. The position of the directional balloon-working channel in the vertebral body does not need to be strictly adjusted, even if the puncture is higher or lower than expected. The expansion direction can be changed by rotating the directional sleeve, without strictly pursuing the puncture direction, which allows for readjustment owing to the unsatisfactory direction of puncture or needle entry, thus reducing the trauma and decreasing operation time. One of the important functions of the balloon is to restore the height of the vertebral body by raising the upper and lower endplates of the diseased vertebrae and to partially correct kyphosis deformities. The directional balloon can reduce collapse endplate and correct the kyphosis deformities more effectively because of the resistance of the working sleeve, as confirmed by clinical results, and suggesting that this technique could also be used in patients who were difficult to perform traditional PKP surgery on, such as those with ruptured and collapsed endplates and severely compressed vertebrae. Follow-up showed that the wedge-shaped angle in group 2 was significantly better than that in group 1 at 3 days after surgery; however, there was no significant difference in the improvement of the wedge-shaped angle and the posterior convex angle between the 2 groups after 3 and 6 months postsurgery. We speculate that the maintenance of the vertebral body height might be related to the progression of osteoporosis, and the directional balloon does not significantly increase the amount of bone cement injected.

Thoracolumbar pain caused by OVCFs is most likely caused by the stimulation of the periosteal nerve by the fretting of the fracture area; therefore, stabilizing the fracture area and restoring the strength of the fracture vertebra are thought to be one of the main mechanisms of VAP to relieve the thoracolumbar pain caused by OVCFs.^[5]^ Therefore, sufficient filling of bone cement in the fracture area is key to relieving the thoracolumbar pain caused by OVCFs.^[27]^ In the traditional PKP procedure, it is difficult to send the balloon to the ideal position. To reduce the incidence of complications, bone cement may not be fully filled in the fracture area. By controlling the expansion direction of the balloon, the bone cement can be filled more fully in the fracture area, thus providing better immediate stability. During the 12-month follow-up, the VAS, ODI, and RMD scores for the patients in group 2 were significantly lower than those in group 1 at 3 days after surgery (e.g., better pain relief and better physical function). In addition, the result showed that the wedge-shaped and kyphosis angles were improved more significantly 3 days after the directional balloon operation, which could be other reasons for this difference in VAS, ODI, and RMD scores, because when the balloon corrected the kyphosis, it could also loosen the pulled posterior branch of the spinal nerve to relieve the pain. Therefore, we believe that the directional balloon technique is more helpful in improving the early outcome of OVCF than the traditional technique, which means that it can relieve the patient's symptoms more quickly.

Although we have demonstrated the advantages of using a directional balloon in the present study, several limitations should be noted. First, in the present study, only the application of the directional balloon technique in single-segment fresh OVCF was studied. However, its application in multiple-stage OVCFs, old compression fractures, and other pathological fractures is unclear. Second, the strength comparison between the controllable directional device and the bone and balloon needs further research. Third, the sample size of the present study was small and the follow-up time was relatively short. Therefore, a larger, longer follow-up randomized controlled study in the future is required. Furthermore, although the prestudy hypothesis loss rate was 10%, it ended up at nearly 20%, which might reduce the credibility of the results. Finally, compared with the traditional balloon dilator, the long-term beneficial effect of this technique remains to be determined.

## 5 Conclusion

The application of a new directional balloon in the treatment of OVCFs not only shortens the operation time and reduces radiation and bone cement leakage, but also improves the early outcome. The directional balloon might be a prominent measure in the treatment of OVCFs, although many mechanisms need to be further studied.

## Acknowledgments

We sincerely thank Professor Zhang Yuan-Yu (Affiliated Baoan Hospital of Shenzhen, Southern Medical University) for his invaluable advice, constant encouragement, and precise modification of the manuscript. We would also like to express our appreciation to all the members who participated in the study, because they provided help in this study. In addition, we also thank nativeee.com for its linguistic assistance during the preparation of this manuscript.

## Author contributions

**Conceptualization:** Pu Wang, Peng Zhan.

**Data curation:** Pu Wang, Jin Li, Zukun Song, Guangye Wang.

**Formal analysis:** Pu Wang, Guangye Wang, Peng Zhan.

**Investigation:** Pu Wang, Zukun Song, Guangye Wang.

**Methodology:** Peng Zhan.

**Project administration:** Peng Zhan.

**Software:** Pu Wang, Jin Li, Zukun Song, Peng Zhan.

**Supervision:** Guangye Wang, Peng Zhan.

**Validation:** Jin Li, Zukun Song, Peng Zhan.

**Visualization:** Jin Li, Zukun Song.

**Writing – original draft:** Pu Wang, Guangye Wang, Peng Zhan.

**Writing – review & editing:** Guangye Wang, Peng Zhan.
